# Histone Deacetylases Inhibit the Snail2-Mediated EMT During Metastasis of Hepatocellular Carcinoma Cells

**DOI:** 10.3389/fcell.2020.00752

**Published:** 2020-08-05

**Authors:** Yue Hu, Qing Nie, Mingrui Dai, Fangfang Chen, Hui Wu

**Affiliations:** ^1^Department of Gastrointestinal and Colorectal Surgery, China-Japan Union Hospital of Jilin University, Changchun, China; ^2^National Engineering Laboratory for AIDS Vaccine, School of Life Sciences, Jilin University, Changchun, China; ^3^Key Laboratory for Molecular Enzymology and Engineering, The Ministry of Education, School of Life Sciences, Jilin University, Changchun, China

**Keywords:** epithelial-mesenchymal transition, Snail2, metastasis, histone deacetylases, hepatocellular carcinoma

## Abstract

Snail2 has an important role in the epithelial-mesenchymal transition (EMT) and tumor metastasis. Here, we report that Snail2 is highly expressed during TGF-β induced EMT in HL-7702 cells. Additionally, overexpression of Snail2 successfully promotes the migration and invasion of these cells, both *in vitro* and in a mouse model. Furthermore, our results show that HDAC1 and HDAC3 could suppress the Snail2 gene promoter. Moreover, we find that the acetylation of H3K4 and H3K56 are significantly reduced during the EMT process of liver HL-7702 cells. Thus, our results indicate that HDAC1 and HDAC3 epigenetically suppress the expression of Snail2 during the EMT of liver cells, revealing an opposing function of HDACs during the migration of malignant tumors.

## Introduction

Worldwide, liver cancer is the seventh most common cancer in 2018, and is the third leading cause of cancer-related deaths (Bray et al., 2018). The resection and transplantation for liver cancer therapies are conventional, but are hampered because of high recurrence rates and the development of metastasis (Forner et al., 2012). Numerous studies have been reported that the epithelial – mesenchymal transition (EMT) is an important step which increased metastasis of tumor cells in breast (Thiery et al., 2009), prostate (Khan et al., 2015), liver (van Zijl et al., 2009), and lung (Soltermann et al., 2008) cancers. Accumulating data suggest that the EMT is an important initiation step for liver cancer metastasis (Nieto et al., 2016). Therefore, to decrease the incidence and mortality rates of liver cancer, prevention of the EMT is critical for inhibiting metastasis.

Regulation of the EMT involves multiple growth factors (e.g., transforming growth factor (TGF), hepatocyte growth factor, and epidermal growth factor) (Said and Williams, 2011) and transcriptional inhibitors (e.g., Snail, ZEB1, and twist) (Díaz-López et al., 2015). In tumor cells, these growth factors and transcriptional inhibitors could regulate EMT by extracellular stimuli derived from the tumor microenvironment. The Snail family includes Snail1, Snail2, and Snail3, that is a group of highly related zinc-finger transcription factors. As transcription factors, they could regulate the EMT and cell migration. Members of this family, particularly Snail1 and Snail2, are functional primarily as repressors of gene transcription, regulating a variety of epithelial-specific genes involved in cell adhesion and epithelial cell identity (Kajita et al., 2004). A previous study has indicated that Snail2 participates in the EMT and tumor metastasis. In human breast tumors, Snail2 and Twist1 promote the EMT and tumor metastasis (Casas et al., 2011). Aberrant expression of JMJD3 could upregulate the expression of Snail2 to promote cancer properties in HCC, such as stem cell-like behaviors and metastasis (Tang et al., 2016).

Several studies have indicated that epigenetic regulation could regulate the gene expression and activation of signaling pathways. The inhibitors of HDACs have been used in the treatment of certain cancers, because histone deacetylases (HDACs) are involved in the metastatic process of cancer (Wang et al., 2018). Stabilization of HDAC1 via the TCL1-pAKT-CHFR axis is a key element for NANOG-mediated multi-resistance and the stem-like phenotype in immune-edited tumor cells (Woo et al., 2018). In addition, HDAC1 promote glioblastoma cell proliferation and invasion via activation of the phosphoinositide 3-kinase/AKT and mitogen-activated protein kinase kinase/extracellular signal-regulated kinase signaling pathways (Li et al., 2018). Moreover, in pancreatic ductal cancer, an HDAC inhibitor suppresses the EMT by targeting Snail1 (Shinke et al., 2018).

In this study, we demonstrate that, among EMT-related transcription repression factors, only Snail2 is significantly upregulated during the TGF-β1–induced EMT in HL-7702 cells. In contrast, silencing of Snail2 promotes the expression of E-cadherin and downregulated the Vimentin expression. Furthermore, in HL-7702 cells, the overexpression of Snail2 induces invasive migration of HL-7702 cells *in vitro* and in the mice model. Notably, HDAC1 and HDAC3 act on the Snail2 promoter to suppress its transcription. Mechanistically, acetylated H3K4 and H3K56 are decreased on the Snail2 promoter during the EMT process. Thus, HDAC1 and HDAC3 epigenetically suppress the expression of Snail2 during the EMT of liver cells. Overall, the present study clarifies a possible signaling pathway and relates molecular mechanisms by which HDAC1 and HDAC3 inhibit the Snail2-mediated EMT during HCC metastasis.

## Materials and Methods

### Viruses and Stable Cell Lines

Lentivirus vector expressing enhanced green fluorescent protein (NC) and lentivirus expressing green fluorescent protein and Snail2 (Snail2) was purchased from Hanbio (China) and used in accordance with standard protocols. Briefly, HL-7702 cells were infected with lentivirus in medium containing polybrene (2 mg/ml) for 24 h and then cultured in RPMI-1640 supplemented with 10% FBS and puromycin (2 μg/ml) at 37°C under 5% CO_2_.

### Antibodies, RNA Interference, and Inhibitors

Antibodies against E-cadherin (20874-1-AP), N-cadherin (22018-1-AP) and GAPDH (60004-1-Ig) were purchased from Proteintech Group (China). The antibody against Snail2 (sc-166476) was from Santa Cruz Biotechnology (United States). Acetylated H3K4 (C15410322) and H3K56 (C15410213) antibodies were purchased from Diagenode.

Transient knockdown experiments were conducted using human siRNA for Snail2, HDAC1, HDAC3, and siControl (Gene Pharma, China). HL-7702 cells were transfected with the indicated siRNAs using Lipofectamine 2000 transfection reagent according to the manufacturer’s protocols. The siRNA sequences as following: siSnail2: 5′-GGA CCA CAG UGG CUC AGA AUU-3′; siHDAC1: 5′ – GCU UCA AUC UAA CUA UCA ATT – 3′; siHDAC3: 5′ – GCA CCC GCA UCG AGA AUC ATT – 3′.

Mocetinostat (S1122) and RGFP966 (S7229) were purchased from Selleck (United States). The concentrations of Mocetinotat and RGF966 were 10mM in luciferase assay and wound healing assay.

### Quantitative Real-Time PCR

Total RNA from tissue samples were isolated with TRIzol reagent (Invitrogen, United States). Quantitative real-time PCR data were analyzed using the comparative Ct method, and expressions of target genes were normalized to that of β-actin.

### Wound Healing Assay

The wound healing assay was performed as analyze the migratory potential of cells as described previously (Liang et al., 2007). HL-7702 cells were seeded in 6-well plates (1^∗^10^6^ cells/well). After 24 h, scrape the cell monolayer in a straight line to create a “scratch” with a p200 pipet tip. Remove the debris and smooth the edge of the scratch by washing the cells once with 1 ml of the growth medium and then replace with 2 ml of 2% fetal bovine serum RPM1-1640. Inhibitors were added to the medium after scratching. The width of the wound was measured under a microscope (Nikon, DS-U2).

### Invasion Assay

The transwell invasion assay was performed as analyze the invasive potential of cells as described previously (Tiwari and Pattnaik, 2017). Cells were counted and 2^∗^10^4^ cells were seeded into cell culture inserts with a pore size of 8.0 μm coated with Matrigel (BD Biosciences). Cells that invaded through the Matrigel-coated membrane after 48 h were fixed with 95% ethanol, and then stained with 0.1% crystal violet. The fold change in invasion was calculated by dividing the number of invading HL-Snail2 cells by the number of invading control cells.

### Liver Metastasis Model

Animal protocols were approved by the Ethical Committee of Care and Use of Laboratory Animals at Jilin University. Female BALB/c nude mice (6–8 weeks old, Charles River, China) for inducing tumorigenesis were injected subcutaneously with HL-7702-N (control) or HL-Snail2 cells (5 × 10^5^cells/mouse; 8mice/group). Visible liver metastatic tissues and tumor tissues were examined and embedded in paraffin, sectioned, and subjected to H&E.

### Luciferase Assay

The Dual-Luciferase Reporter Assay System (Promega, United States) was used as described previously (Hossan et al., 2016). HL-7702 cells were co-transfected with a Snail2 promoter containing the luciferase construct (pGL3-Bisic, Promega) together with a plasmid expressing Renilla luciferase. Firefly luciferase activity was normalized to Renilla luciferase activity to control for transfection efficiency.

### Chromatin Immunoprecipitation (ChIP) Assay

Chromatin immunoprecipitation assays were performed according to the protocol described previously (Boyer et al., 2006). One 10 cm dish (1 × 10^7^) of HL-7702 N and HL-7702 Snail2 cells were grown to 90% of confluence and used for each ChIP assay. IgG was used as a negative control. ChIP assay was conducted essentially as described using H3K4ac and H3K56ac antibodies. ChIP DNA was subjected to qPCR. Both ChIP and IgG-antibody signals were normalized to the total input. The primers for the Snail2 promoter were: 5′-TAGCTCCCAGAGAGCGTGGA -3′ and 5′-AGGTTCAGATTTCAGCTCCTCC -3′.

### Statistical Analyses

Results are represented as mean ± SD. Significant differences between two groups were assessed using 2-tailed Student’s *t*-test. A *p*-value of ≤0.05 was considered statistically significant. *p* ≤0.05, ≤0.01, and ≤0.001 are represented by single, double and triple asterisks respectively.

## Results

### The TGF-β–Induced EMT Requires Snail2 in Liver Cells

**FIGURE 1:**
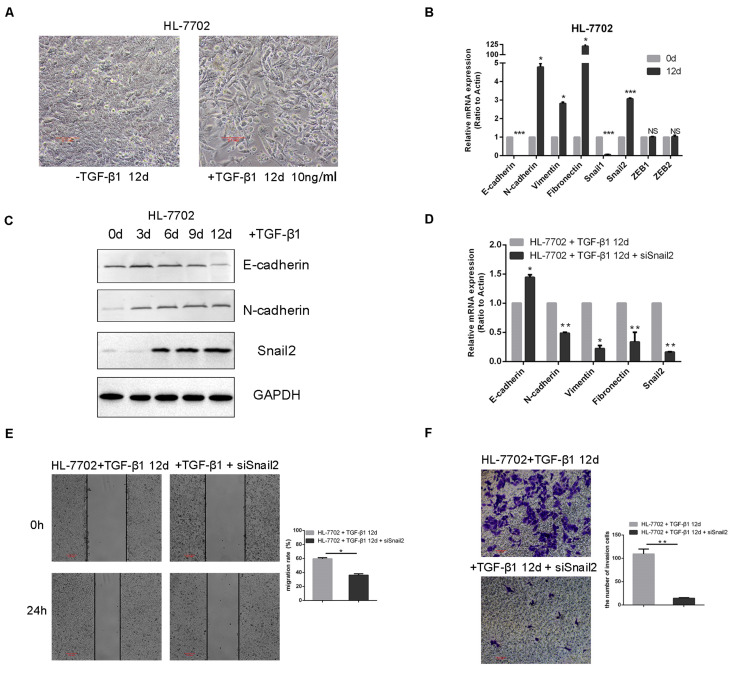
TGF-β–induced EMT requires Snail2 in liver cell. **(A)** HL-7702 liver cells were treated with TGF-β1 (10 ng/ml) for 12 days. Cell morphological changes associated with the EMT are shown in the phase contrast images. The scale bar was 0.1 mm. **(B)** HL-7702 cells were treated with TGF-β1 (10 ng/ml) for 12 days, then analyzed by Relative mRNA levels are shown as means ± SD. *P ≤ 0.05; ***P ≤ 0.001. **(C)** HL-7702 cells were treated with TGF-β1 (10 ng/ml) for 0, 3, 6, 9, and 12 days, then analyzed by western blotting. **(D)** SiRNA of Snail2 (siSnail2) was expressed in HL-7702 after treating with TGF-β1 (10 ng/ml) for 12 days and the expression of EMT markers were determined by qRT-PCR. **(E,F)** The abilities of migration and invasion of HL-7702 cells treating with TGF-β1 (10 ng/ml) for 12 days or transfected siSnail2 were analyzed in wound healing and invasion assays. Data in the histogram were shown as means ± SD from three independent experiments. The scale bar was 0.2 mm. A representative experiment was shown. **P ≤ 0.01.

To identify transcription factors during the TGF-β–induced EMT, we explored the time and concentration of TGF-β1 required for the EMT in HL-7702 cells (Supplementary Figure S1). Based on morphological changes (i.e., cells undergo the dissolution of tight junctions, the destruction of apically basal polarity, and the recombination of cytoskeleton structures) which enable cells to develop an invasive phenotype during the EMT, we confirmed that the optimal concentration of TGF-β1 was 10 ng/ml and induction time was 12 days (Figure 1A). As expected, TGF-β1 treatment resulted in the induction of Snail2, the acquisition of a fibroblastic mesenchymal morphology, downregulation of an epithelial marker (E-cadherin), and upregulation of mesenchymal markers (N-cadherin, vimentin, and fibronectin) in HL-7702 cells (Figures 1B,C). Furthermore, when we knocked-down the expression of Snail2 in HL-7702 cells after treating TGF-β1 12 days, the expression of E-cadherin increased and the expression of N-cadherin, fibronectin, vimentin were significantly decreased (Figure 1D). In Figures 1E,F, we treated cells with TGF-β1 for 12 days and then transfected siSnail2; the results showed that abilities of migration and invasion were suppressed when Snail2 was knockdown. Thus, Snail2 is necessary for the TGF-β–induced EMT process of liver cells.

### Snail2 Promotes Migration and Metastasis in HCC Cells

**FIGURE 2:**
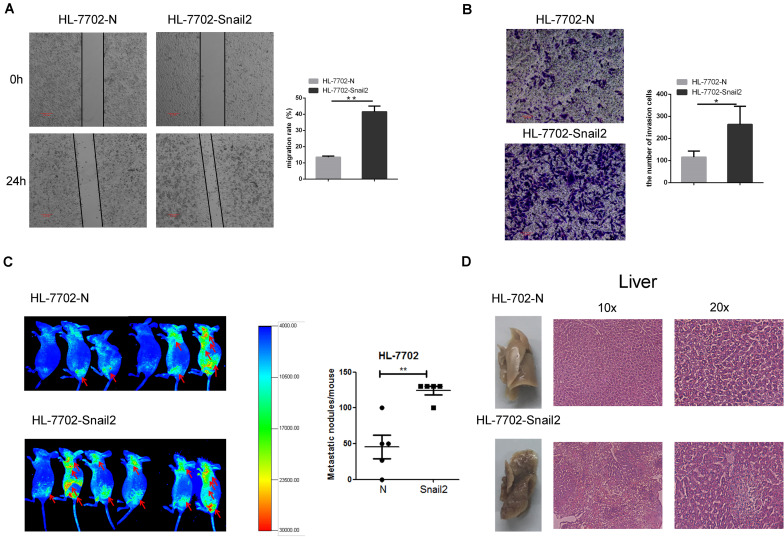
Snail2 promotes migration and metastasis of HL-7702 cells. **(A,B)** The abilities of migration and invasion of HL-7702 cells (overexpressing Snail2 or control) were analyzed in wound healing and invasion assays. The scale bar was 0.2 mm. Data in the histogram were shown as means ± SD from three independent experiments. **(C)** HL-7702 cells (overexpressing Snail2 or control) were injected subcutaneously into nude mice. The green fluorescent protein signal was detected using an *in vivo* imaging system at 30 days. The numbers of mice with distant metastasis at 30 days after injection of Snail2-overexpressing or control HL-7702 cells was shown. **(D)** After 30 days, eight mice per group were sacrificed and livers were dissected. Liver metastatic nodules were examined macroscopically, or paraffin-embedded, cut into sections, and stained with hematoxylin and eosin. *P ≤ 0.05, **P ≤ 0.01.

To further test whether Snail2 could regulate the migratory and invasive abilities of HL-7702 cells, we established the Snail2 overexpression cell line (Supplementary Figure S2). Wound healing assay assessed the effect of Snail2 on cell migration. Snail2 overexpression cells (HL-7702-Snail2) had significantly higher migration compared with control cells (Figure 2A). Moreover, Snail2 overexpression cells (HL-7702-Snail2) showed a greater rate of invasion (Figure 2B).

To verify the migratory and invasive capacities *in vivo*, we investigated whether Snail2 altered the tumorigenic properties of liver cells. HL-7702-Snail2 and control cells were injected subcutaneously into nude mice. Using an *in vivo* imaging system to detect the migration of tumor cells in real time, we found that mice in the Snail2 group showed a stronger green fluorescent protein signal than the control group (labeled the comparison area with arrows), and the Snail2 overexpression mice had large numbers of liver metastases compare with control group (Figure 2C). This suggested that Snail2 could promote metastasis of HCC cells *in vivo*. 30 days later, we narcotized and dissected the mice. We found that the livers of mice injected with HL-7702-Snail2 cells underwent metastases, and even were necrotic. Hematoxylin and eosin staining showed that the liver cell arrangement of mice injected with HL-7702-Snail2 cells was irregular, no contour was detected and there was unclear distinction between the nucleus and cytoplasm (Figure 2D).

### HDAC1 and HDAC3 Suppress Snail2 Transcription Through Deacetylation of H3K56 and H3K4

**FIGURE 3:**
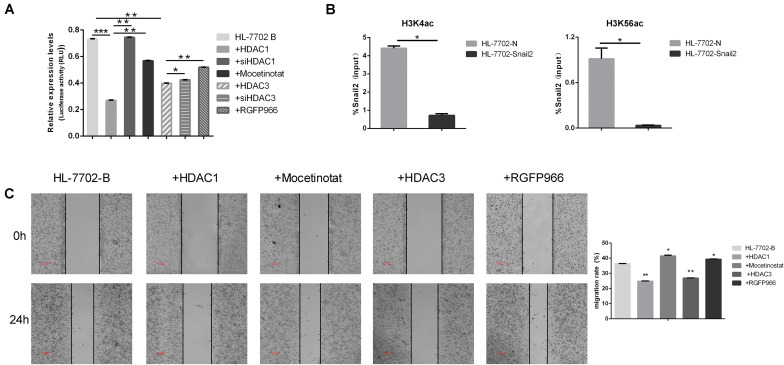
HDAC1 and HDAC3 repress Snail2 expression through deacetylation of H3K56 and H3K4. **(A)** HL-7702 cells were created to express the luciferase reporter plasmid, pGL3-Snail2 promoter-Luc. The cells were then transfected with the pGL3-Snail2 promoter -Luc vector and plasmids of HDAC1, HDAC3, siHDAC1, or siHDAC3. Mocetinostat or RGFO966 were added to the cell growth medium and, 24 h later, luciferase activity was assayed and normalized to that of Renilla luciferase (pRL-SV40), which served as an internal control. Each data point represents the mean ± SD. *P ≤ 0.05; **P ≤ 0.01; ***P ≤ 0.001. Experiments were performed twice in triplicate. **(B)** Acetylated H3K4 and H3K56 at the Snail2 promoter were analyzed by chromatin immunoprecipitation (ChIP) (upper panel). ChIP samples were also analyzed by the quantitative real-time polymerase chain reaction (means ± SD from three separate experiments; bottom panel). **(C)** HL-7702 cells were transfected with the HDAC1 or HDAC3 plasmids. Mocetinostat and RGFO966 were added to the cell growth medium for 24 h. The cells were then analyzed in the wound healing assay. The scale bar was 0.2 mm. Data in the histogram are shown as means ± SD from three independent experiments.

Several results indicated that epigenetic factors were involved in the regulation of EMT transcription inhibitors. We next further examined the potential role of HDACs family in controlling Snail2 promoter activity using the dual luciferase reporter assay. As shown in Figure 3A, co-transfection of HDAC1 or HDAC3 expression vector led to a dramatic reduction in Snail2 promoter activity as compared to vector-transfected control cells. On the contrary, we co-transfection of siHDAC1 or siHDAC3 expression vector led to upregulate in Snail2 promoter activity as compared to vector-transfected HDACs cells. Moreover, we treated HL-7702 cells transfected with the pGL3-Snail2-Luc vector with Mocetinostat or RGFP966, which specifically inhibit HDAC1 and HDAC3, respectively. Upon treating cells with the inhibitors, luciferase activity recovered and was even greater than HDACs overexpression, suggesting the direct suppression of Snail2 by HDAC1 and HDAC3 (Figure 3A).

To confirm the direct repression of Snail2 by HDACs, we investigated the acetylation level on the Snail2 promoter by the chromatin immunoprecipitation assay using stable Snail2-overexpressing cells. Acetylated H3K56 and H3K4 are the main targets of HDAC1 and HDAC3. As shown in Figure 3B, acetylated H3K4 and H3K56 were reduced at the Snail2 promoter in HL-Snail2 compared with control cells. Our results indicated that the promoter region of Snail2 was deacetylated by HDAC1 and HDAC3 during the EMT process in HCC cells.

Finally, we investigated whether HDAC1 and HDAC3 could influence the migration of HL-7702 cells. As shown in Figure 3C, compared with control HL-7702 cells, overexpression of HDAC1 and HDAC3 significantly decreased migration. In contrast, treatment with HDAC1 and HDAC3 inhibitors restored the migration capacity of these cells to the normal level. Taken together, these data indicate that HDAC1 and HDAC3 may inhibit Snail2-mediated transcriptional repression through histone deacetylation.

## Discussion

Triggering the EMT upregulates a core of transcription factors, including Snail, Twist, and ZEB, those repress the EMT related marker, E-cadherin, and ultimately coordinate the EMT process (Diaz-Lopez et al., 2014). In our study, we confirmed that the expression of Snail2 was increased during the TGF-β–induced EMT in HL-7702 cells. Knockdown the expression of Snail2 upregulated the expression of E-cadherin and downregulated the expression of N-cadherin, fibronectin and vimentin, while overexpression of Snail2 induced invasive migration of liver cells *in vitro and in vivo*.

A wide range of genetic and epigenetic modifications play pivotal roles in the development and tumorigenesis of cancer (Yi et al., 2014). These epigenetic changes are associated with DNA methylation and histone modifications. Moreover, there are numerous papers reporting that HDACs can promote the progression of cancer (Wu et al., 2011; Zhang et al., 2019). In breast cancer, SREBP1 regulates the EMT by forming a co-repressor complex with HDAC1/2 and Snail1 to suppress E-cadherin and promote tumor metastasis (Zhang et al., 2019). Another study reported that nardilysin controls intestinal tumorigenesis through HDAC1/p53-dependent transcriptional regulation. In addition, interplay between HDAC3 and WDR5 is essential for the hypoxia-induced EMT (Wu et al., 2011). Recently, several HDAC inhibitors have been approved by the United States Food and Drug Administration as anticancer drugs (Garnock-Jones, 2015). These include Vorinostat and Romidepsin that have anticancer activity against cutaneous T-cell lymphoma. However, we found that HDAC1 and HDAC3 acted on the Snail2 promoter to suppress the transcription of Snail2. We also demonstrated that acetylated H3K56 and H3K4, which are targets of HDAC1 and HDAC3 respectively, were reduced at the Snail2 promoter in EMT model cells. Above all, our results suggested that HDAC1 and HDAC3 suppressed the expression of Snail2 through the deacetylation of H3K56 and H3K4, triggering the repression of the Snail2-mediated EMT.

**FIGURE 4:**
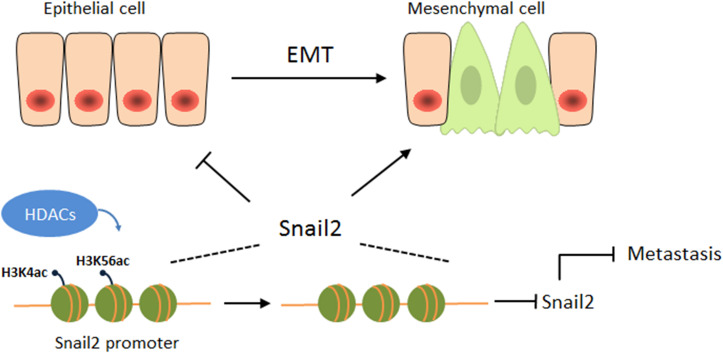
HDACs suppress the expression of Snail2 during the EMT. A proposed working model of HDAC1 and HDAC3 epigenetically suppress the expression of Snail2 during the EMT of liver cells, revealing an opposing function of HDACs during the migration of HCC.

Overall, this study is the first to reveal an opposing function of HDACs during the migration of hepatocellular carcinoma (HCC) (Figure 4). HDACs may play different roles in different cancers, even in the same cancer, and may be involved in different pathways, eventually leading to different outcomes. Our study also emphasizes the attention of the possibility of HDAC inhibitors as anticancer drugs for HCCs.

## Data Availability Statement

The raw data supporting the conclusions of this article will be made available by the authors, without undue reservation, to any qualified researcher.

## Ethics Statement

The animal study was reviewed and approved by the Animal Experimental Platform, Core Facilities for Life Science, Jilin University.

## Author Contributions

YH designed the experiments and wrote the manuscript. YH, QN, and MD performed the experiments and obtained the data. All authors contributed to the article and approved the submitted version.

## Conflict of Interest

The authors declare that the research was conducted in the absence of any commercial or financial relationships that could be construed as a potential conflict of interest.
