# Supplementary material for: Histone Deacetylases Inhibit the Snail2-Mediated EMT During Metastasis of Hepatocellular Carcinoma Cells
Source: Front Cell Dev Biol. 2020 Aug 5;8:752. doi: 10.3389/fcell.2020.00752 (PMC7419474; doi:10.3389/fcell.2020.00752)
Supplement: Supplementary file 1 [file Data_Sheet_1.PDF]

Figure S1.

The liver cell line HL -7702 was treated with TGF-β1 for 0, 3, 6, 9, 12 days and the concentration of TGF-β1 was 10 ng/ml or 20 ng/ml; cell morphological changes associated with EMT are shown as phase contrast images. The scale bar was 0.1mm.

Figure S2.

(A) The efficiency of lentivirus infected HL-7702 detected by fluorescence microscopy. The scale bar was 0.2mm. (B) The expressions of HDAC1, HDAC3, and Snail2 in HL-7702-N and HL-7702-Snail2 cell lines were analyzed by Western blotting.
