# Supplementary figures and images for: Histone Deacetylases Inhibit the Snail2-Mediated EMT During Metastasis of Hepatocellular Carcinoma Cells
Source: Front Cell Dev Biol. 2020 Aug 5;8:752. doi: 10.3389/fcell.2020.00752 (PMC7419474; doi:10.3389/fcell.2020.00752)

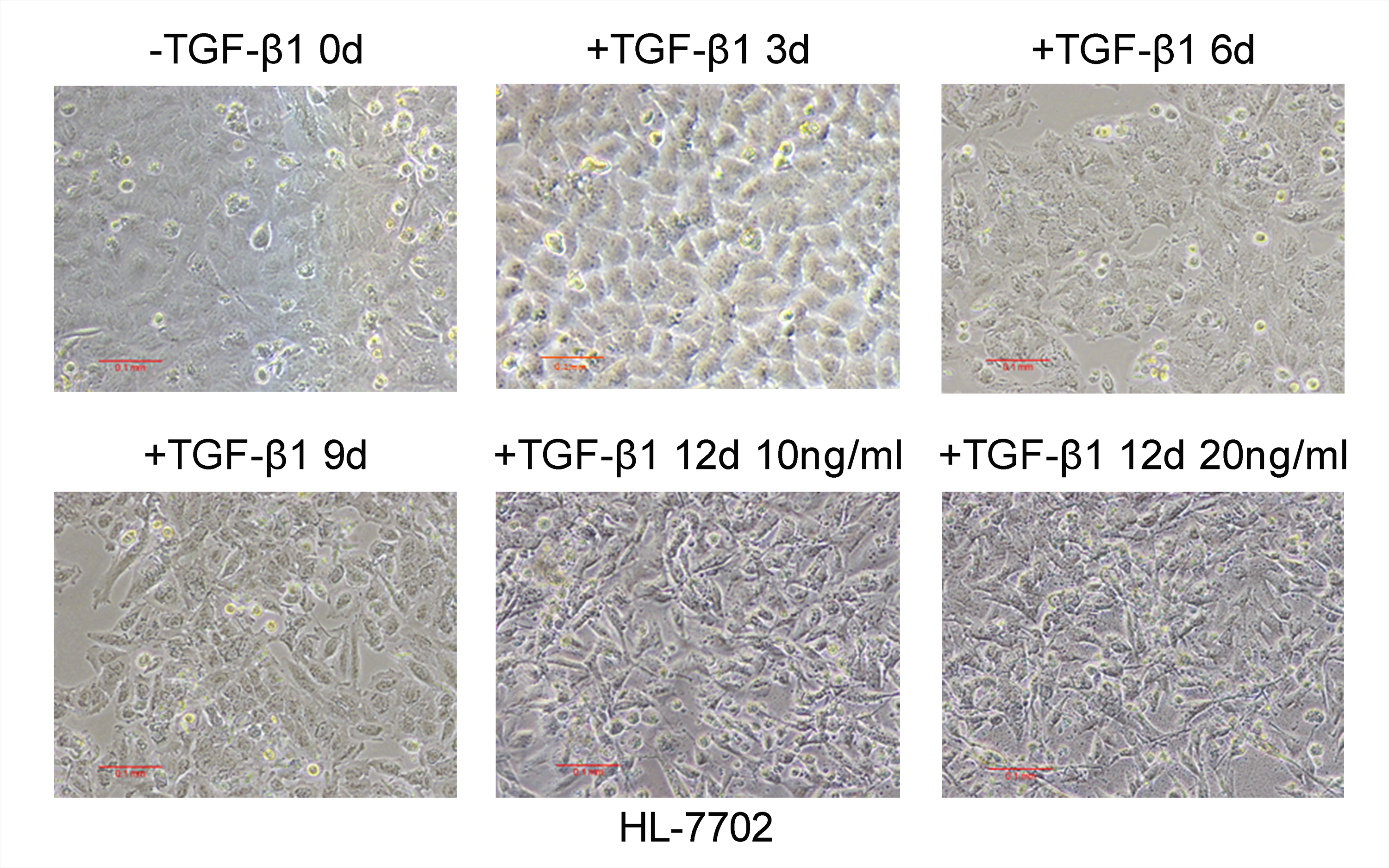

Supplement: Supplementary file 2 [file Image_1.TIF]

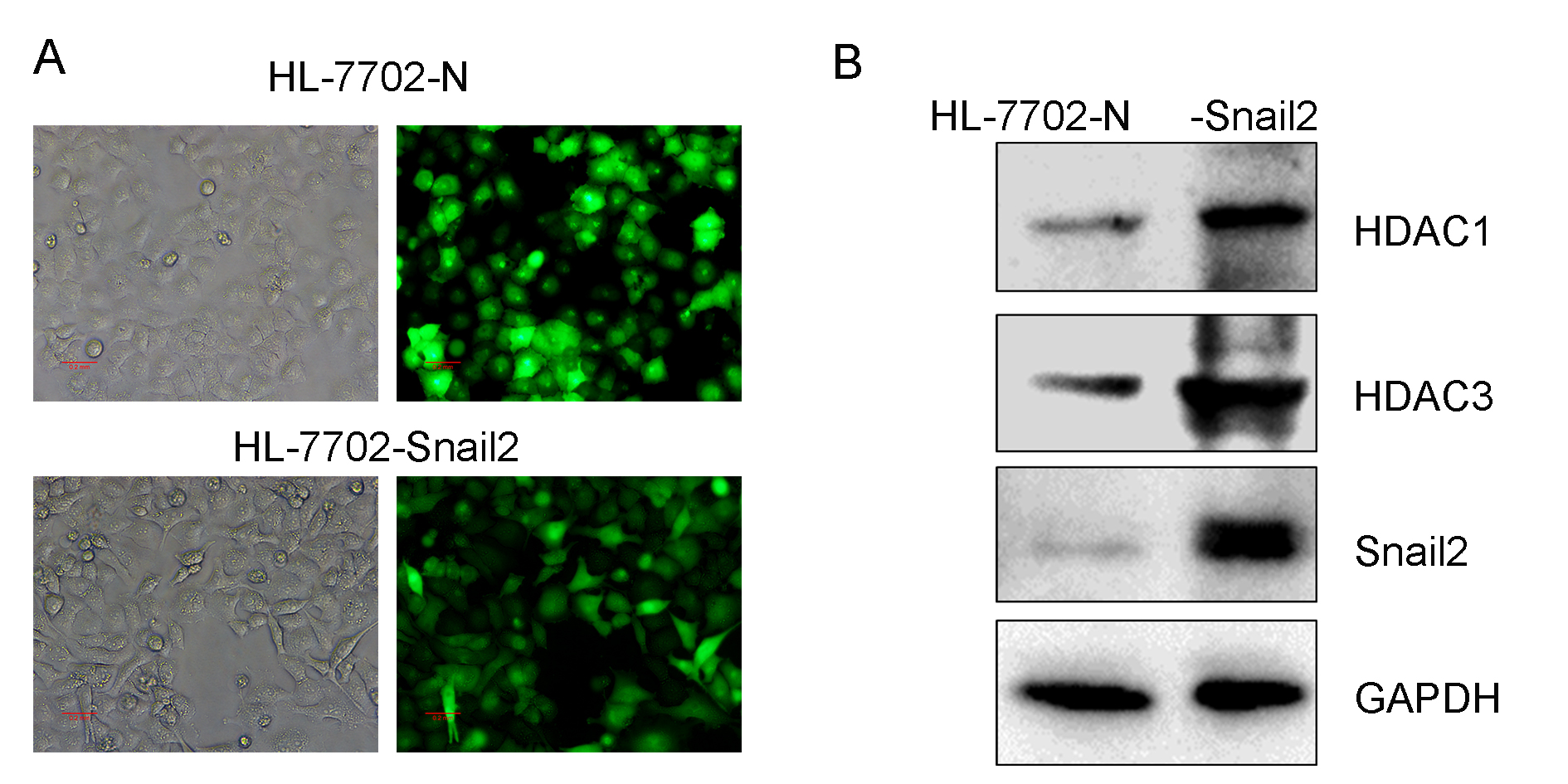

Supplement: Supplementary file 3 [file Image_2.TIF]
